# Trafficking of activated lymphocytes into the RENCA tumour microcirculation in vivo in mice.

**DOI:** 10.1038/bjc.1997.599

**Published:** 1997

**Authors:** N. J. Brown, S. Ali, M. W. Reed, R. Wiltrout, R. C. Rees

**Affiliations:** Department of Surgical and Anaesthetic Sciences, University of Sheffield, Royal Hallamshire Hospital, UK.

## Abstract

The aim of the study was to establish a model of tumour microcirculation in vivo using the murine renal cell carcinoma cell line (RENCA) implanted into the mouse cremaster muscle, and subsequently to investigate the trafficking of syngeneic lymphocyte subpopulations into both the RENCA tumour and the surrounding normal cremaster muscle microcirculation. We have demonstrated that RENCA tumour cells, at a dose of 1.5 x 10(5) per 30 microl injected into the cremaster muscle, reproducibly produced a vascularized tumour suitable for in vivo microscopy at 10-14 days. Injection of fluorescently labelled effector cells (1 x 10(6)) including naive splenocytes, T-cell enriched populations and ex vivo interleukin 2 (IL-2)-activated splenocytes all migrated to and flowed through both the tumour and the normal microcirculation, with negligible adhesion. However, we observed the selective recruitment, localization and arrest of IL-2-activated splenocytes (P < 0.05) into the tumour microcirculation, and the subsequent extravasation of cells into the tumour intestitium in some instances. This did not occur with the other effector cells. We also observed the absence of leucocyte rolling in the tumour microcirculation, suggesting an impairment in adhesion molecule expression on the tumour endothelium. We have therefore established the potential of this model for defining further effector cell-tumour-endothelium interactions.


*British Journal of Cancer* (1997) **76**(12), 1572–1578
© 1997 Cancer Research Campaign

### Trafficking of activated lymphocytes into the RENCA tumour microcirculation in vivo in mice

**NJ Brown[1], S Ali[2], MWR Reed[1], R Wiltrout[3] and RC Rees[2]**

[1]Department of Surgical and Anaesthetic Sciences, University of Sheffield, Floor K, Royal Hallamshire Hospital, Glossop Road, Sheffield S10 2JF; [2]Institute for Cancer Studies, The Medical School, Beech Hill Road, Sheffield S10 2RX, UK; and [3]Laboratory of Experimental Immunology & Biological Response Modifiers Programme, National Cancer Institute, Frederick, ML, USA

**Summary** The aim of the study was to establish a model of tumour microcirculation in vivo using the murine renal cell carcinoma cell line (RENCA) implanted into the mouse cremaster muscle, and subsequently to investigate the trafficking of syngeneic lymphocyte subpopulations into both the RENCA tumour and the surrounding normal cremaster muscle microcirculation. We have demonstrated that RENCA tumour cells, at a dose of $1.5 \times 10^5$ per 30 μl injected into the cremaster muscle, reproducibly produced a vascularized tumour suitable for in vivo microscopy at 10–14 days. Injection of fluorescently labelled effector cells ($1 \times 10^6$) including naive splenocytes, T-cell enriched populations and ex vivo interleukin 2 (IL-2)-activated splenocytes all migrated to and flowed through both the tumour and the normal microcirculation, with negligible adhesion. However, we observed the selective recruitment, localization and arrest of IL-2-activated splenocytes ($P < 0.05$) into the tumour microcirculation, and the subsequent extravasation of cells into the tumour intestitium in some instances. This did not occur with the other effector cells. We also observed the absence of leucocyte rolling in the tumour microcirculation, suggesting an impairment in adhesion molecule expression on the tumour endothelium. We have therefore established the potential of this model for defining further effector cell–tumour–endothelium interactions.

**Keywords:** leucocyte/lymphocyte trafficking; tumour microcirculation; RENCA tumour; immunotherapy

Adoptive immunotherapy using cytokines alone or in combination with activated lymphocytes, such as lymphokine-activated killer (LAK) cells, has been demonstrated to produce tumour necrosis and a dramatic reduction in the number of metastases in a variety of animal models (Ettinghausen and Rosenberg, 1986; Lafreniere et al, 1988; Schwarz et al, 1988). The clinical use of adoptive immunotherapy has been successful in the treatment of some advanced cancers, in particular malignant melanoma and renal cell carcinoma. Regimens using interleukin 2 (IL-2) in combination with LAK cells demonstrated an overall response rate of 33% in patients with renal cell carcinoma and 23% in patients with melanoma (Rosenberg et al, 1987; Hayatt et al, 1991). However, a much larger study involving 327 patients with advanced renal cell carcinoma failed to show an increase in overall response rate when comparing LAK + IL-2 (18%) with IL-2 alone (15%; Palmer et al, 1992). Adoptive immunotherapy using tumour-infiltrating lymphocytes (TILs) in combination with IL-2 demonstrated a 40% overall response rate in patients with renal cell carcinoma (Rosenberg et al, 1988).

Systemic administration of rhIL-2 alone (Rosenberg et al, 1987) in combination with LAK cells promotes the in vivo proliferation of LAK cells in the lungs of mice and causes tumour regression (Ettinghausen et al, 1985). The limited numbers of patients responding to this therapy and the potentially serious side-effects of high-dose cytokine therapy (such as vascular leak syndrome) highlight the need to perform preclinical studies in vitro and in vivo to increase our understanding of the mechanisms involved in activated lymphocyte-induced anti-cancer responses. Lymphocyte migration via the microcirculation to the site of the tumour is a prerequisite for a therapeutic response, thus, assessment of the ability of lymphocyte subsets to migrate to and localize within the tumour may allow improved efficacy of this therapeutic approach.

Until recently, it has been difficult to assess accurately the migration and behaviour of adoptively transferred effector cells in vivo. The technique of in vivo microscopy permits dynamic visualization of fluorescently labelled effector cells (Brown and Reed, 1997; Sasaki et al, 1991). Cells can be visualized moving through the microcirculation, migrating across the endothelium and basement membrane and localizing within the tumour. This technique is therefore an effective method of monitoring leucocyte trafficking in vivo. Only one study has quantified the in vivo migration and distribution of fluorescently labelled human adherent-LAK (A-LAK) cells into the VX2 carcinoma in the rabbit ear chamber model using in vivo microscopy (Sasaki et al, 1991). A small number of A-LAK cells preferentially accumulated in the tumour microcirculation, but no extravasation was observed. Adhesion of A-LAK cells within the tumour vasculature appeared to damage endothelial cells, and cessation of tumour blood flow occurred 48 h after administration, followed by tumour necrosis and a diffuse infiltration of lymphocytes, monocytes and granulocytes into the tumour interstitial space. The occurrence of tumour necrosis, despite a low effector to target cell ratio, suggested that a decreased tumour blood flow starved the tumour of nutrients and oxygen and that the direct cytotoxicity played a minimal role. However, this is a xenogenic model, using human lymphocytes and a rabbit tumour system, and interpretation of the significance of these observations may have limitations.

*Received 25 October 1996*
*Revised 14 May 1997*
*Accepted 23 May 1997*

*Correspondence to:* NJ Brown

The present study aimed to (a) establish a model of tumour microcirculation in vivo using the murine renal cell carcinoma cell line (RENCA) implanted into the mouse cremaster muscle and (b) investigate the trafficking of syngeneic lymphocyte subpopulations in both the tumour and host microcirculation using fluorescent in vivo microscopy (IVM). The model used in these studies is in vivo microscopy of the mouse cremaster preparation (Brown and Reed, 1997), modified from the original technique described in the rat (Baez, 1973). We have observed the selective recruitment and localization of IL-2-activated splenocytes into the tumour microcirculation, and established the potential of this model for defining further effector cell, tumour endothelial interactions.

### MATERIALS AND METHODS

### Animals

Experiments were initially performed on 6-week-old Balb/c mice weighing 15 g, obtained from Sheffield Field Laboratories, UK. All experiments were approved by the Home Office and performed within project licence number PPL 50/0695.

### RENCA tumour cells

The RENCA tumour (renal cortical adenocarcinoma) arose spontaneously in Balb/c mice and was isolated by Dr S Stewart at the National Cancer Institute Bethesda, ML, USA with growth and progression after transfer of as few as 50 viable cells (Murphy and Hruskesky, 1980). The immunogenicity of RENCA has been determined as being low to moderate. The RENCA tumour becomes highly vascularized as it develops and its progressive growth and spontaneous metastasis from the kidney occurs in a similar manner to that generally described for human renal cell cancer (Bassil et al, 1985).

The RENCA cell line was maintained by in vitro passage in RPMI-1640 medium containing 1% fetal calf serum (FCS), 1% sodium pyruvate, 10% NEAA and maintained at 37°C in a humidified atmosphere of 5% carbon dioxide in air. The cell line was routinely checked to ensure freedom from mycoplasma (Mycoplasma rapid detection system, Gena-Probe, USA).

### Preparation of spleen cells (splenocytes)

The spleens from Balb/c mice were removed aseptically and transferred to a sterile, plastic Petri dish. Using a 5-ml syringe and 23-G needle, the spleens were ruptured and the cells dislodged gently by flushing them out of the spleen with 10 ml of RPMI-1640 medium. This process was repeated until only the connective tissue remnants remained. Red blood cells (RBCs) were lysed by the addition of 5 ml of RBC lysis buffer (ammonium chloride, 8.3 mg ml$^{-1}$; potassium bicarbonate, 1 mg ml$^{-1}$; and EDTA, 37 µg ml$^{-1}$ in distilled water) to the splenocyte pellet. The cells were then washed twice in RPMI-1640 medium containing FCS, counted and resuspended ready for use.

### Fractionation of splenocytes

Nylon wool columns were prepared by packaging 0.6 g of dried nylon wool (Division off Travenol Laboratories, Thetford, Norfolk, UK) into 10-ml syringe barrels. The column was saturated with RPMI-1640 medium containing 10% FCS and incubated at 37°C in 5% carbon dioxide in air for 45 min. An aliquot of 1–2 × 10$^{7}$ splenocytes in 2 ml of medium was loaded onto each column and incubated again for 45 min. Non-adherent (enriched T lymphocytes) cells were eluted slowly from the column in 20–30 ml of prewarmed medium, washed twice and resuspended to the required concentration. Unfractionated and nylon wool column eluted splenocytes contained 28.5 ± 6.8% and 48.1 ± 6.5% Thy 1-positive T lymphocytes respectively, measured using flow cytometric analysis.

**Table 1** Flow cytometric analysis of leucocyte subpopulations and integrin expression after in vitro culture with or without the addition of rhIL-2 (mean ± s.e.m.)

| Splenocytes | Antibody | IL-2 activated ($n$ = 4) | Non-IL-2 activated ($n$ = 4) |
|---|---|---|---|
| T cells | MAS108cs (Thy 1) | 31.6 ± 6.4% | 21.8 ± 4.6% |
| CD4 cells | FMAS 110p (L3/L4) | 17 ± 1.4% | 21.8 ± 2.7% |
| CD8 cells | FMAS111c (Lyt-2) | 12.9 ± 4% | 11.7 ± 2.7% |
| Macrophages | VTF-034-10 | 0.5% | 0.4% |
| Integrin | CD11a | | |
| Expression | | 276.6 ± 21% | 95 ± 8.3% |

Splenocytes (2 × 10$^{6}$ cells ml$^{-1}$) from normal animals (8–10 weeks) were cultured in the presence or absence of rhIL-2 at a concentration of 1000 U ml$^{-1}$ in 24-well plates for 4 days. Cells were then harvested, washed and phenotyped with a panel of antibodies (see Materials and methods).

### Preparation of in vitro activated (LAK) cells

Splenocytes were resuspended in RPMI-1640 medium supplemented with 10% FCS, 2 mM glutamine and 5 × 10$^{-5}$ 2-mercaptoethanol at a concentration of 2 × 10$^{-6}$ cells ml$^{-1}$. Cells were placed in a 24-well flat-bottomed tissue culture plate, 1 ml per well, recombinant interleukin 2 (rhIL-2; 1000 U ml$^{-1}$; Glaxo, UK) was added to each well and the plates cultured for 4 days at 37°C and 5% carbon dioxide and 95% air to induce effector cells mediating LAK activity. The cells were then removed from the wells, washed twice and resuspended in complete medium to be fluorescently labelled for the in vivo microscopy experiments or to use in the cytotoxicity assays.

LAK cells are large granular lymphocytes, 95–100% expressing a natural killer (NK) cell phenotype. The percentage of CD4 and CD8 positive T cells and macrophages was shown to be similar for splenocytes cultured in the presence or absence of rhIL-2. However, a 31% decrease in the number of Thy 1-positive cells was observed when cultured in the absence of IL-2 (Table 1).

### Flow cytometric assay

The following antibodies were purchased from Sera-Lab (Crawly Down, Sussex, UK) and used at the manufacturers' recommended concentrations for the identification of leucocyte subpopulations and integrin expression. MAS 108cs (Thy-1), FMAS 111c (Lyt-2), FMAS 250 (anti-bromodeoxyuridin, used as a negative control), VTF-034-10 (macrophages), FMAS 110p (L3/L4, helper/inducer), FMAS 112p (anti-rat IgG2b) and CD11a (rat anti-mouse, Serotech, Oxford, UK). Samples were analysed on a Becton Dickinson FACSort (Oxford, UK) and generally 10 000 cells were acquired with appropriate forward (FSC) and side (SSC) scatter to confirm lymphocyte identity. The percentage and relative intensity of FITC

© Cancer Research Campaign 1997

Ab binding was recorded on the whole (ungated) and lymphocyte gated populations using 930/30 nm bandpass filter for fluorescence.

### Tumour implantation

Animals were anaesthetized with an intraperitoneal injection of diazepam (0.5 mg $ml^{-1}$, Dumex Risborough, UK) and Hypnorm (fentanyl citrate 0.0315 mg $ml^{-1}$ and fluanisone 1 mg $ml^{-1}$, Janssen Pharmaceutical Oxford, UK) in the ratio of 1:1 at a volume of 0.1 ml per 200 g body weight, with supplementation as required to maintain adequate anaesthesia. An abdominal incision was made and the testis was pulled up into the abdominal cavity via the testicular ligament, exposing the inner surface of the cremaster muscle. RENCA tumour cells ($1.5 \times 10^5$ cells per 30 µl) were injected into the cremaster muscle. Mice were then allowed to recover and used for in vivo microscopy 10–14 days later, at which time the tumour had become vascularized.

### Surgical procedure for in vivo microscopy

Tumour-bearing mice were anaesthetized using the protocol described in the previous section. A midline incision was made in the neck and a tracheostomy was performed. The left carotid artery was cannulated and connected to a pressure transducer and physiograph (Micro-Med, Louisville, KY, USA) to monitor mean arterial blood pressure and heart rate. The cannula also provided access for the administration of fluorescently labelled cells. An oesophageal thermistor probe was inserted and connected to a thermometer (Fluke, Washington, USA). The animal was then placed on a warming pad to maintain body temperature (35–37°C). Another thermistor was placed between the animal and the warming pad to prevent over-heating. The cremaster muscle, with intact neurovascular supply was prepared for in vivo microscopy, as described in detail in a previous publication (Brown and Reed, 1997).

### In vivo microscopy

The animal was transferred to the stage of a Nikon fluorescence microscope (Orthophot) equipped with a tungsten lamp for transmitted light microscopy and a mercury arc lamp for epi-illumination fluorescent light microscopy. A filter cube interposed into the light path of the mercury arc lamp permitted blue (430–470 nm) light to be selected for epi-illumination. Images of the preparation were monitored using a silicon-intensified tube camera (SIT, Hamammatsu Phototonics, UK), displayed on a high resolution monitor (Sony PVM-1443) and recorded on video (Sony SLV-373-UB) tape for later off-line analysis.

After transferring the preparation to the microscope, a further thermistor was placed under the edge of the cremaster and connected to the thermometer. All instruments were calibrated before each experiment. The animal was allowed 30 min to equilibrate before experimentation; temperature and blood pressure were monitored at 5-min intervals, initially, and then every 15 min for the remainder of the experiment.

### Fluorescent labelling of effector cells for in vivo microscopy

Cells ($1 \times 10^7$ $ml^{-1}$) were incubated for 15 min at 37°C with 5 µl of the fluorochrome bis carboxy-ethyl-carboxy-fluorescein acetoxymethyl ester (BCECF-AM 3 µM) in medium. Cells were washed twice and resuspended in 1 ml of medium. BCECF-AM does not affect cell viability as assessed by trypan blue cell proliferation using [$^3$H]thymidine uptake (Garbett et al, 1994).

### Experimental protocol

Animals were divided into four groups ($n$ = 5 in each group). During the equilibration period, areas of interest were selected within the cremaster muscle and the tumour, which could be clearly visualized using transmitted or fluorescent light, and used to estimate the numbers of lymphocytes interacting with both the tumour and the normal microcirculation. After the equilibration period, 0.1 ml of labelled cells ($1 \times 10^6$) were injected via the carotid cannula. The following subpopulations were assessed: (a) splenocytes, (b) nylon wool, non-adherent splenocytes (enriched T lymphocytes), (c) in vitro IL-2-activated lymphocytes from the spleen, and (d) in vitro unactivated lymphocytes (–IL-2) from the spleen as controls.

### Data collection and image analysis

Areas of the normal cremaster muscle were selected to estimate that the numbers of lymphocytes within the vessels had normal blood flow and could be clearly visualized using transmitted and fluorescent light. The vessels studied were arterioles in the range 10–30 µm, venules in the range 30–40 µm and post-capillary venules < 5 µm. In the tumour it is difficult to categorize the vessels, therefore an area of interest was chosen containing vessels with diameters similar to those in the normal cremaster muscle.

Lymphocytes entering the microcirculation of the normal cremaster muscle or the RENCA tumour were subdivided into three categories:

a. no contact with the vessel wall – flying;
b. adherent to but moving along the vessel wall – rolling;
c. adherent and stationary within the vessel for more than 30 s – adherent.

Measurements were taken for 1 min every 10 min for the 2-h duration of the study. Vessel diameters were measured using computerized image analysis calibrated to produce values in microns and vessel flow was assessed qualitatively. Numbers of fluorescently labelled effector cells within each area of interest were counted during each minute of recording.

### Statistical analysis

Numbers of cells per 250 µm vessel length per minute were expressed as median and range. The Wilcoxon signed rank test for non-parametric data was used to analyse paired data (within group comparison) and an ANOVA test was performed for between group comparisons. Results were considered statistically significant at $P < 0.05$.

### RESULTS

### Characteristics of the normal and tumour microcirculation

Tumour cells at a dose of $1.5 \times 10^5$ cells per 30 µl injected into the cremaster muscle reproducibly produced a vascularized tumour of a size 0.2–0.4 $mm^3$ suitable for IVM after 10–14 days (data not shown). The normal cremaster muscle microcirculation is an organized network of vessels consisting of arterioles, capillaries,

© Cancer Research Campaign 1997

**Table 2** Frequency of flying cells (median and range) within the RENCA tumour and the normal cremaster microcirculation.

| | 0 min | | 60 min | | 120 min | |
|---|---|---|---|---|---|---|
| | T | N | T | N | T | N |
| Splenocytes | 40<br>30–60 | 32<br>20–45 | 30<br>15–35 | 25<br>15–30 | 25*<br>10–30 | 15*<br>8–18 |
| T lymphocytes | 35<br>25–55 | 30<br>20–40 | 22<br>6–27 | 15<br>10–20 | 20*<br>15–30 | 10*<br>9–20 |
| IL-2 activated[a] | 20<br>12–40 | 8<br>6–20 | 15<br>8–20 | 10<br>5–14 | 18<br>10–20 | 12<br>0–20 |
| IL-2 activated[b] control | 24<br>15–28 | 10<br>9–20 | 18<br>10–22 | 12<br>8–18 | 15<br>8–24 | 10<br>0–12 |

Data, median and range; T, tumour microcirculation; N, normal microcirculation; *$P < 0.05$ vs numbers of cells at time 0 min using the non-parametric Wilcoxon test for within group comparison. [a]Splenocytes incubated for 4 days at 37°C in a 5% carbon dioxide atmosphere in RPMI medium supplemented with 10% fetal calf serum, $5 \times 10^{-5}$ 2 ME and 1000 U ml$^{-1}$ rhIL-2. [b]Splenocytes incubated as above but in the absence of rhIL-2.

**Table 3** Frequency of adherent cells (median and range) within the RENCA tumour and the normal cremaster microcirculation

| | 0 min | | 60 min | | 120 min | |
|---|---|---|---|---|---|---|
| | T | N | T | N | T | N |
| Splenocytes | 1<br>0–1 | 1<br>0–2 | 1<br>0–2 | 1<br>0–1 | 1<br>0–2 | 1<br>0–1 |
| T lymphocytes | 2<br>0–2 | 1<br>0–1 | 1<br>0–2 | 1<br>0–1 | 0<br>0–1 | 0<br>0–1 |
| IL-2 activated[a] | 10*<br>6–20 | 1<br>0–1 | 15*<br>8–20 | 0<br>0–1 | 12*<br>6–14 | 1<br>0–1 |
| IL-2 activated[b] control | 1<br>0–1 | 2<br>0–2 | 2<br>0–2 | 2<br>0–2 | 1<br>0–2 | 1<br>0–1 |

Data, median and range; T, tumour microcirculation; N, normal microcirculation; *$P < 0.05$ vs control (no rhIL-2) using non-parametric ANOVA for between group comparison. [a]Splenocytes incubated for 4 days at 37°C in a 5% carbon dioxide atmosphere in RPMI medium supplemented with 10% fetal calf serum, $5 \times 10^{-5}$ 2 ME and 1000 U ml$^{-1}$ rhIL-2. [b]Splenocytes incubated as above but in the absence of rhIL-2.

**Figure 1** Comparison of flying (**A**) and adherent (**B**) rhIL-2 activated lymphocytes in the tumour microcirculation (⊠) and the normal microcirculation (□) at the beginning (0 min) and the end (120 min) of the experiment. Data are represented as median and range above the bars with $n = 5$ for each bar. *Indicates a statistically significant difference in lymphocyte frequency between tumour and normal vessels ($P < 0.05$)

venules and post-capillary venules. These can be identified according to diameter and direction of blood flow. In contrast, the RENCA tumour microcirculation is highly disorganized with an increased number of vessels/high power field, which are difficult to identify because of their convoluted nature. They have numerous bifurcations and blind ends and in the smaller tumour vessels bidirectional flow as well as interrupted flow was observed.

After injection of the fluorescently labelled effector cells ($1 \times 10^6$), the number of cells entering the visual field in 1 min at the beginning of the experiment was greater than the number entering the same field at the end of the experiment for all subpopulations of effector cells (Table 2). More effector cells entered the visual field in the tumour microcirculation compared with the normal microcirculation (Table 2). In the normal microcirculation, the blood vessel diameters used for assessing lymphocyte interactions were 30–40 μm for the venules, 10–30 μm for the arterioles and < 5 μm for the capillaries. An area in the tumour was chosen for study, with vessels ranging up to 40 μm. Blood flow was assessed qualitatively, and in some areas of the tumour it appeared to be greater than in the normal microcirculation.

### Trafficking studies

After injection of $1 \times 10^6$ fluorescently labelled effector cells into tumour-bearing mice, labelled cells could be visualized moving through both the tumour and the normal microcirculation. This allowed analysis of the number of cells adhering, rolling and flying within the areas of interest.

The frequency of flying cells ranged from 12 to 60 cells in the tumour microcirculation and from 6 to 45 in the normal microcirculation at the beginning of the experiment. There was a decrease in the numbers of cells flying in both the tumour (8–30) and the normal (0–20) microcirculation by the end of the experiment (120 min), suggesting that the cells were being trapped somewhere within other microcirculatory beds, probably in the lungs (Table 2).

No rolling cells were observed in the tumour microcirculation with any of the effector populations used in this study. However, only a few cells (1–3) from each of the effector populations were capable of rolling in the normal microcirculation at all time points monitored.

© Cancer Research Campaign 1997

Naive splenocytes or T-cell-enriched populations, although able to traffic through the tumour and normal microcirculation, did not adhere to the endothelium. Apart from the reduction in the number of Thy 1-positive cells, the functionally distinct, IL-2-activated splenocytes demonstrated the same proportion of $CD4^+$ and $CD8^+$ lymphocytes and macrophages as control cultured spleen cells (Table 1). However IL-2-activated effectors demonstrated a two- to threefold increase in fluorescent intensity in the presence of the CD11a antibody when compared with controls (splenocytes), indicating increased integrin expression. On injection into the tumour-bearing mice, the number of IL-2-activated cells adhering within the RENCA tumour microcirculation was significantly greater (tumour vs normal; 10 vs 1; $P < 0.05$) when compared with the control cultured splenocytes (Table 3). Neither population was adherent to the normal endothelium (Table 3). Readings taken at 0, 60 and 120 min were consistent, demonstrating that even after 2 h, IL-2-activated cells were still able to adhere to the endothelium. The localization of the cells was heterogeneous throughout all of the tumours studied. The activated lymphocytes did not adhere within particular areas of the tumour or in vessels of a particular diameter, however, lymphocytes did adhere in clusters. It was observed in two out of five experiments that some of the IL-2-activated cells adhering to the tumour endothelium at the beginning of the study had extravasated into the tumour intestitium by 2 h. The frequency of IL-2-activated splenocytes (flying and adherent) entering either the tumour or the normal microcirculation is shown in Figure 1.

### Physiological parameters

The heart rate, blood pressure and body temperature of all animals remained constant throughout the experimental period. Mean arterial pressure was 100 ± 19 mmHg, and the mean pulse rate was 467 ± 50 beats per min. Body temperature, as measured by the oesophageal thermocouple, was within the range 36.3–37.2°C. Cremaster temperature measured with a thermocouple positioned at the edge of the muscle was within the physiological range.

### DISCUSSION

Previous studies have investigated the mechanisms of lymphocyte-mediated anti-tumour responses in vitro, but until recently it has been difficult to accurately assess the migration and behaviour of adoptively transferred effector cells in vivo (Sasaki et al, 1991). Initial studies of leucocyte and tumour cell migration used radiolabelled effector cells and removal of the organ as assessment of radioactivity at a specified time after cell administration. However, although these studies demonstrated increased migration of cells into the liver and spleen it was thought to be due to non-specific uptake of released radiolabel into the organ (Wiltrout et al, 1983; Basse et al, 1990). These studies allowed quantification of cell migration but provided no information on cell behaviour and dynamics in vivo. Fluorescent dyes have now become available that are non-toxic to cells and allow the direct visualization of injected effector cells, providing more accurate estimation of the cell distribution into tissues.

Basse and his colleagues (1992) compared in vivo cell migration of radiolabelled and fluorescently labelled A-LAK cells, by either counting radioactivity or the number of fluorescent cells in frozen tissue sections and concluded that the latter provided a more accurate index of A-LAK migration. Again, this study provided valuable information on cell distribution and migration but no information on cell dynamics.

The technique of in vivo microscopy permits the dynamic visualization of fluorescently labelled effector cells and is an effective method of monitoring lymphocyte trafficking in vivo. A study by Sasaki et al (1991) quantified the in vivo distribution of A-LAK cells and observed tumour necrosis, despite a low effector to target cell ratio. However, this was a xenogenic model using human LAK cells in a rabbit tumour implanted into the rabbit ear, thus, the significance of these observations may be limited. A subsequent paper by the same authors used a syngeneic model to investigate the migration and localization of murine activated natural killer (NK) cells into a murine mammary carcinoma grown in a cranial window preparation (Melder et al, 1995). They demonstrated heterogeneous accumulation of large numbers of adherent NK (A-NK) cells within the tumour microcirculation but only a few cells adhering within the normal pial vessels, and suggested adhesion-mediated retention of the cells within the tumour, but mechanical entrapment of the rigid activated cells within the normal microcirculation.

The results from the present study demonstrated that all the subpopulations of effector cells migrated to the tumour but only effector cells activated in vitro with IL-2 interacted with and adhered to the RENCA tumour endothelium. There was a dramatic and immediate increase in the localization of IL-2-activated splenocytes within 30 s of systemic injection of the cells. The adherent lymphocytes remained localized within the tumour for the duration of the study, with further cells adhering as the study progressed. However, the localization of the cells was heterogeneous throughout all of the tumours studied. The activated lymphocytes did not adhere within particular areas of the tumour or in vessels of a particular diameter, however lymphocytes did adhere in clusters. Some lymphocytes appeared to extravasate through the endothelium into the tumour intestitium. These observations are in agreement with other studies showing heterogeneous but specific localization of activated lymphocytes in tumour vasculature using both xenogenic (Sasaki et al, 1991) and syngeneic models (Basse et al, 1991; Fukumura et al, 1995; Melder et al, 1995).

The mechanism by which LAK cells are initially attracted to the tumour site is not fully understood. They may gain access to the tumour via the microcirculation simply as circulating cells, and adhere because of their activated status, or possibly respond to the additional influence of chemoattractant agents released from within the tumour environment, such as chemokines and cytokines. Although LAK cells mediate an increased level of cytotoxicity, this is not tumour specific (Bergers et al, 1994). Once LAK cells have entered the tumour environment, it is unclear whether they mediate anti-tumour activity through direct lymphocytotoxicity, indirectly by the release of cytokines and the recruitment of additional effector cells or by interaction with and possible destruction of the tumour microcirculation.

A number of factors may enhance the localization of activated lymphocytes within the RENCA tumour environment. These include the architecture of the tumour vasculature (Jain, 1988), the structure and rigidity of in vitro activated lymphocytes (Sasaki et al, 1989) and the expression of adhesion molecules, for example CD11, CD18 and CD2, on the lymphocyte surface (Melder et al, 1990).

Areas within the RENCA tumour possessing bidirectional as well as interrupted flow may have an increased resistance to flow, which may increase the opportunity for lymphocytes to interact with the tumour endothelium. Also, the increase in rigidity of the activated

 © Cancer Research Campaign 1997

cells will obstruct their passage through smaller capillaries, thus increasing the chance of cells lodging within the vessel. In other areas of the tumour, the larger vessels appear to have an increased blood flow (qualitative observation). Thus, the architecture and possible increase in blood flow may explain the increased number of effector cells entering the tumour microcirculation compared with the host microcirculation.

Lymphocytes activated by IL-2 have an increased expression of adhesion molecules on their cell surface, including integrin subunits and LFA-2 (Melder et al, 1990), and this was confirmed in the present study using flow cytometry. It is unknown whether lymphocytes can recognize specific sites of attachment on the tumour endothelium or whether their attachment is simply physical. At the present time, a specific site of attachment identified for activated cells on tumour endothelium has not been demonstrated. There is a heterogeneous pattern of activated lymphocyte adhesion within the tumour microcirculation, but no adhesion within the normal vessels. However, one observation from our studies is that effector cells within the tumour microcirculation do not roll along the endothelium, a phenomenon well characterized in normal vasculature including the cremaster muscle and during inflammation. This may be due to the disorganized flow characteristics of the tumour enabling firm adhesion without initial rolling. The adhesion molecules P-selectin, E-selectin, L-selectin (CD62P and L) and VCAM-1 are all involved in regulating in vivo leucocyte rolling and adhesion (Ley et al, 1995). As rolling of effector cells was not observed in our studies, it is possible that there is down-regulation of adhesion molecules on the tumour endothelium, and this is currently being investigated in our model. Previous studies using a mammary adenocarcinoma implanted into the dorsal skinfold chamber in the rat demonstrated reduced leucocyte adherence and rolling in the tumour microcirculation compared with the normal microcirculation (Wu et al, 1992). This was suggested to be related to a down-regulation in expression of the adhesion molecules ICAM-1 and ICAM-2 and the selectins on the tumour endothelium (Wu et al, 1992), although these studies did not directly confirm this.

Thus, the architecture and blood flow within the tumour, the rigidity of the lymphocytes and the increase in adhesion molecule expression on the lymphocyte cell surface probably contribute to and may determine the trafficking localization of lymphocytes through the tumour microcirculation in our in vivo model.

In conclusion, IL-2-activated murine lymphocytes (lymphokine-activated killer LAK cells) demonstrate a degree of selectivity for adhesion to the murine RENCA tumour microcirculation and possess an increased ability to distribute and localize within the tumour microcirculation. However, because of their shape and rigidity, and the heterogeneous blood flow within a developing tumour, these effector cells do not have the ability to reach all areas of the tumour, which may in part explain why variability in the response of solid tumours to adoptive immunotherapy occurs.

In future studies this model could be used to determine:

a. the propensity of different specifically sensitized, non-specifically activated and quiescent leucocyte populations to migrate to and arrest within the tumour;
b. the specific adhesion molecules involved in the lymphocytes arresting within the tumour using specific antibodies;
c. whether RENCA cells transfected with cytokine genes can regulate the localization and adhesion of lymphocyte subpopulations following systemic delivery.

### ACKNOWLEDGEMENT

We wish to thank the Yorkshire Research Campaign for supporting this work.

### REFERENCES

Baez J (1973) An open cremaster preparation for the study of blood vessels by in-vivo microscopy. *Microvasc Res* **5:** 384–395

Basse PH, Hokland P and Hokland ME (1990) Comparison between $^{125}$IudR and $^{51}$Cr as cell labels investigations of tumour cell migration. *Nucl Med Biol* **17:** 781–791

Basse PH, Nanmark U, Johansson BR, Herberman RB and Goldfarb RH (1991) Establishment of cell-to-cell contacts by adoptively transferred adherent lymphokine activated killer cells with metastatic melanoma cells. *J Natl Cancer Inst* **83:** 944–950

Basse P, Herberman RB, Hokland M and Goldfarb RH (1992) Tissue distribution of adoptively transferred adherent lymphokine-activated killer cells assessed by different cell labels. *Cancer Immunol Immunother* **34:** 221–227

Bassil B, Dosoretz DE and Prout Jr GR (1985) Validation of the tumour, nodes and metastasis classification of renal cell carcinoma. *J Urol* **134:** 450–454

Bergers J, Otter W, Dullens H, Groot J, Steerenberg P, Filius P and Crommelin J (1994) Effect of immunomodulators on specific tumour immunity induced by liposome-encapsulated tumour associated antigens. *Int J Cancer* **56:** 721–726

Brown NJ and Reed MWR (1997) Leucocyte interactions with the mouse cremaster muscle microcirculation in vivo in response to tumour conditioned medium. *Br J Cancer* **75:** 993–999

Ettinghausen SE, Lipford EH, Mule JJ and Rosenberg SA (1985) Recombinant interleukin-2 stimulates in vivo proliferation of adoptively transferred lymphokine-activated killer (LAK) cells. *J Immunol* **135:** 3623–3628

Ettinghausen SE and Rosenberg SA (1986) Immunotherapy of murine sarcomas using lymphokine activated killer cells: optimisation of the schedule and route of administration of recombinant interleukin-2. *Cancer Res* **46:** 2784–2789

Fukumura D, Salehi HA, Witwer B, Tuma RF, Melder RJ and Jain RK (1995) Tumour necrosis factor induced leucocyte adhesion in normal and tumour vessels: effect of tumour type, transplantation site and host strain. *Cancer Res* **55:** 4824–4829

Garbett EA, Reed MWR and Brown NJ (1994) Viability and visualisation of fluorescently labelled lymphocytes in vitro and in vivo. *Int J Micro Clin Exp* **14:** 249

Hayatt K, Rodgers S, Bruce L, Rees RC, Chapman K, Reeder S, Dorren MS, Sheridan E, Sreenivasan T and Hancock BW (1991) Malignant melanoma and renal cell carcinoma: Immunological and haematological effects of recombinant interleukin 2. *Eur J Cancer* **27:** 1009–1014

Jain RK (1988) Determinants of blood flow: A review. *Cancer Res* **48:** 2641–2658

Lafreniere R and Rosenberg SA (1988) Successful immunotherapy of murine experimental hepatic metastases with lymphokine activated killer cells and recombinant interleukin-2. *Cancer Res* **45:** 3735–3741

Ley K, Bullard DC, Arbones ML, Bosse R, Vestweber D, Tedder TF and Beaudet AL (1995) Sequential contribution of L- and P-selectin to leucocyte rolling in vivo. *J Exp Med* **181:** 669–675

Melder RJ, Walker ER, Herberman RB and Whiteside TL (1990) Surface characteristics, morphology and ultrastructure of human adherent lymphokine-activated killer (A-LAK) cells. *J Leukocyte Biol* **48:** 163–173

Melder RJ, Salehi HA and Jain RK (1995) Interaction of activated natural killer cells with normal and tumour vessels in cranial windows in mice. *Microvasc Res* **50:** 35–44

Murphy GP and Hruskesky WJ (1980) A murine renal cell carcinoma. *J Natl Cancer Inst* **50:** 1013–1015

Palmer PA, Vinke J, Evers P, Pourreau C, Oskram R, Roest G, Vlems F, Becker L, Loriaux E and Franks CR (1992) Continuous infusion of recombinant interleukin-2 with or without autologous lymphokine activated killer cells or the treatment of advanced renal cell carcinoma. *Eur J Cancer* **28A:** 1038–1044

Reed MWR, Weiman TJ, Shuschke DA, Tseng MT and Miller FN (1989) A comparison of the effects of photodynamic therapy on normal tumour vessels in the rat microcirculation. *Radiat Res* **119:** 542–552

Rosenberg SA, Lotze MT, Muul LM, Chang AE, Avis FP, Leitman S, Linehan LM, Robertson CN, Lee RE, Rubin JT, Sepp CA, Simpson CG and White DE (1987) A progress report on the treatment of 157 patients with advanced cancer using lymphokine activated killer cells and interleukin-2 or interleukin-2 alone. *N Engl J Med* **316:** 889–897

© Cancer Research Campaign 1997

Rosenberg SA, Packard BS and Aebersold PM (1988) Use of tumour infiltrating lymphocytes and IL2 in immunotherapy of patients with metastatic melanoma. *N Engl J Med* **319:** 1676–1680

Sasaki A, Jain RK, Maghazachi AA, Goldfarb RH and Herberman RB (1989) Low deformability of lymphokine activated killer cells as a possible determinant of in vivo distribution. *Cancer Res* **49:** 3742–3746

Sasaki A, Melder RJ, Whiteside TL, Herberman RB and Jain RK (1991) Preferential localisation of human adherent lymphokine-activated killer cells in tumour microcirculation. *J Natl Cancer Inst* **83:** 433–437

Schwarz RE, Vujanovic NL and Hiserodt JC (1989) Enhanced antimetastatic activity of lymphokine activated killer cells purified and expanded by their adherence to plastic. *Cancer Res* **49:** 1441–1446

Wiltrout RH, Gorelik E, Brunda MJ, Holden HT and Herberman RB (1983) Assessment of in vivo natural antitumour resistance and lymphocyte migration in mice: comparison of $^{125}$I iododeoxyuridine with $^{111}$indium-oxine and $^{51}$chromium as cell labels. *Cancer Immunol Immunother* **14:** 172–179

NZ Klitzman B, Dodge R and Dewhirst MW (1992) Diminished leukocyte-endothelium interaction with tumor microvessels. *Cancer Res* **52:** 4265–4268

*© Cancer Research Campaign 1997*


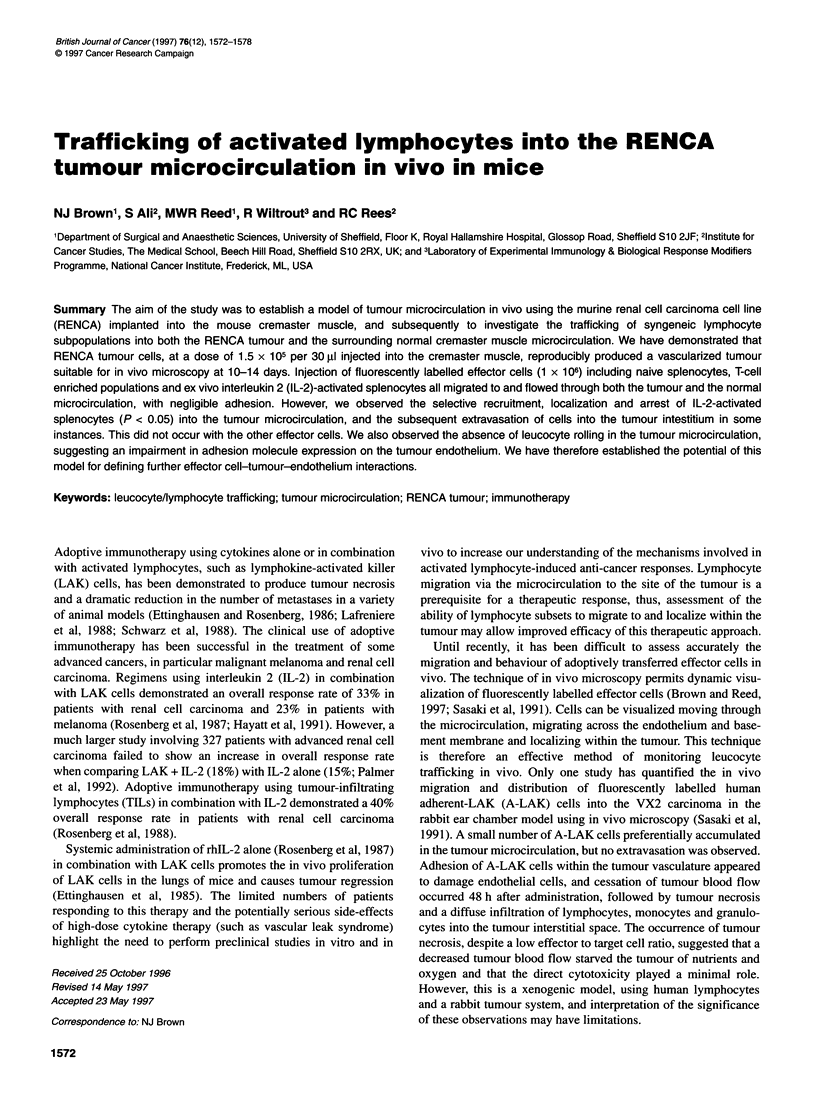

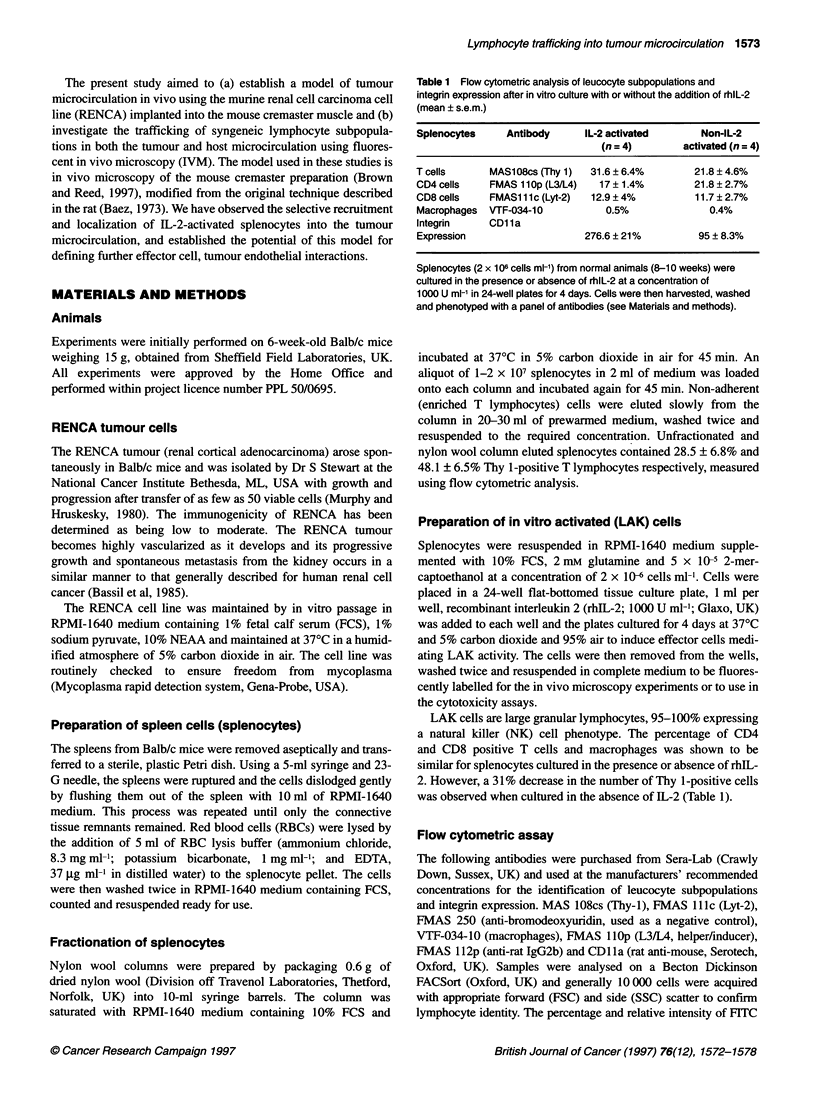

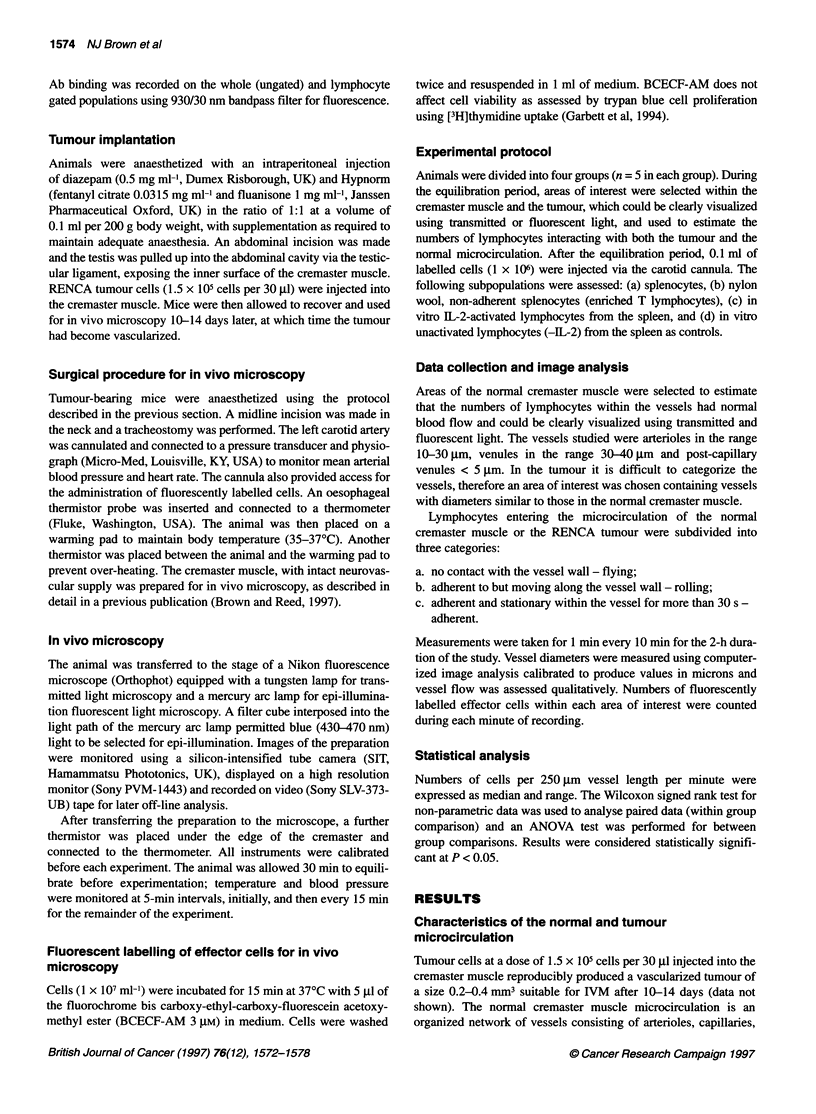

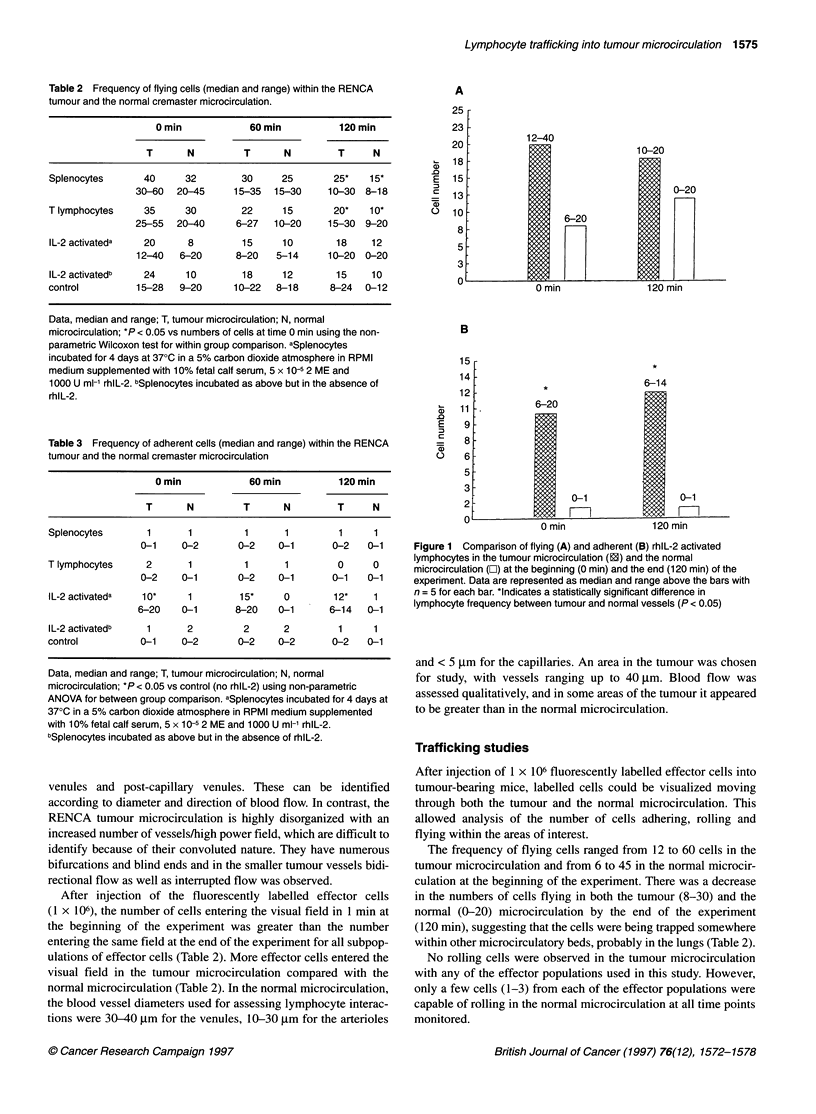

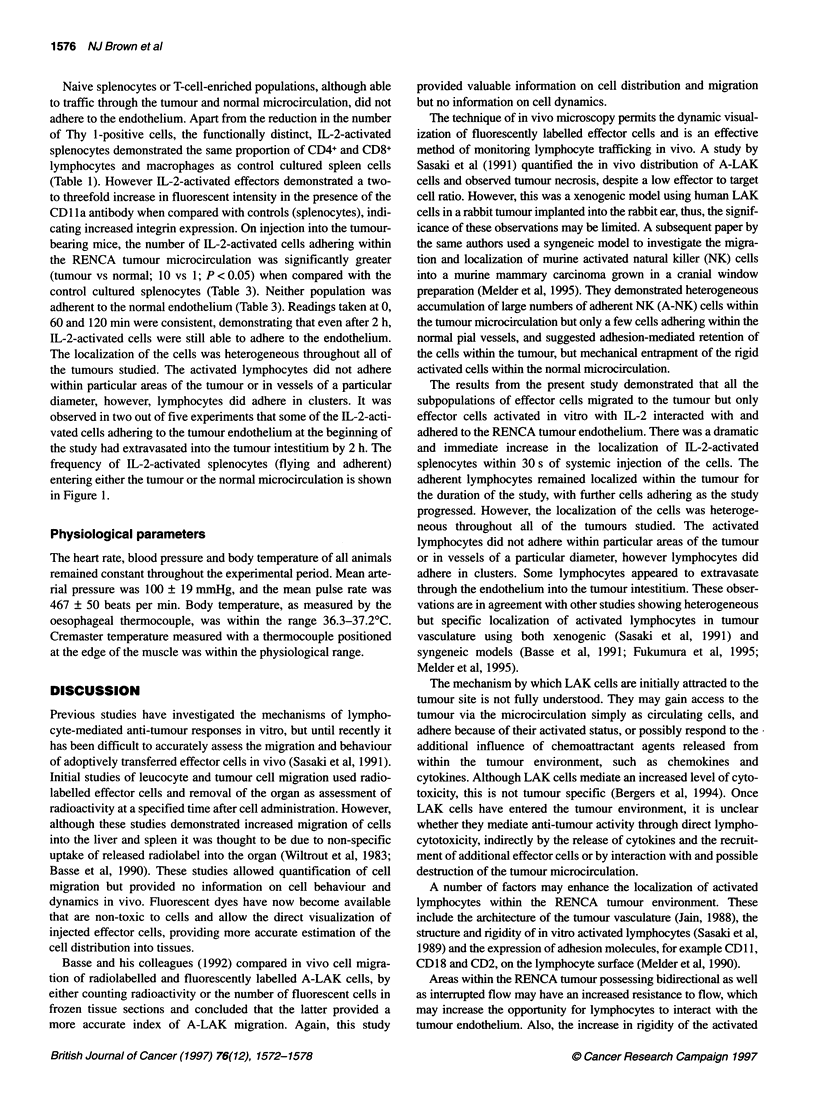

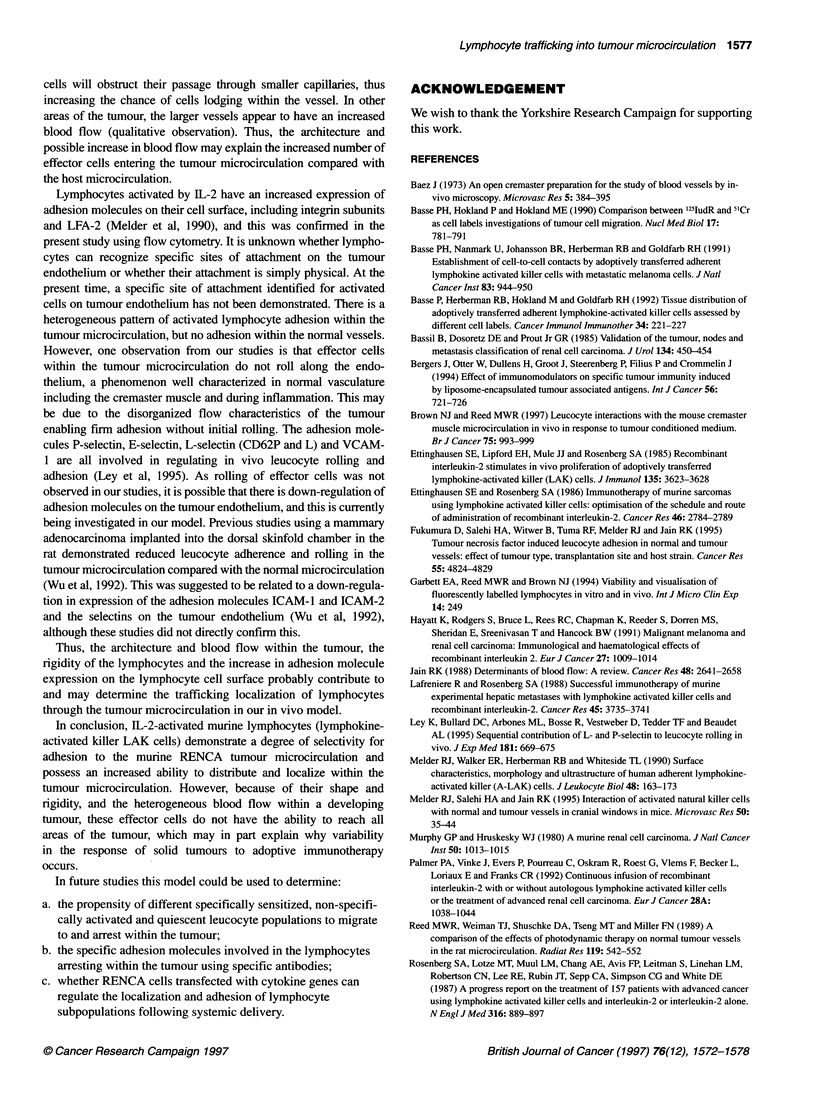

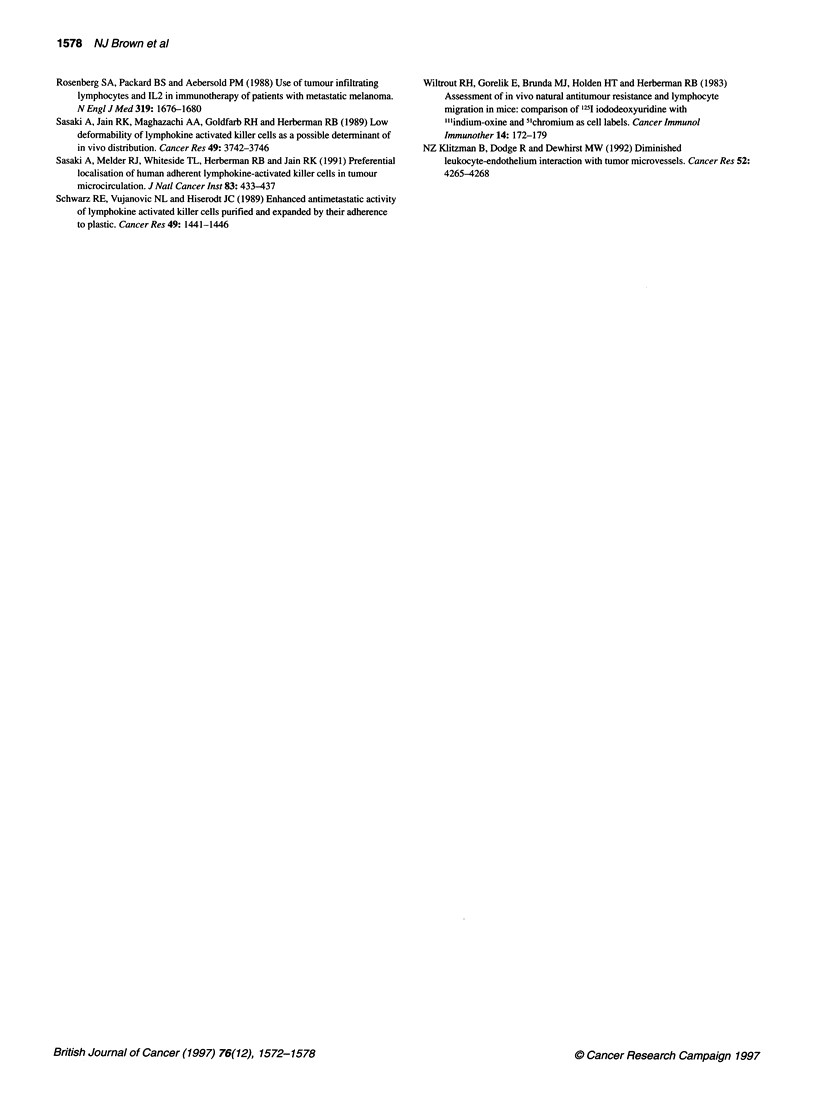


## References

[OCR_00732] Baez S. (1973). An open cremaster muscle preparation for the study of blood vessels by in vivo microscopy.. Microvasc Res.

[OCR_00741] Basse P. H., Nannmark U., Johansson B. R., Herberman R. B., Goldfarb R. H. (1991). Establishment of cell-to-cell contact by adoptively transferred adherent lymphokine-activated killer cells with metastatic murine melanoma cells.. J Natl Cancer Inst.

[OCR_00747] Basse P., Herberman R. B., Hokland M., Goldfarb R. H. (1992). Tissue distribution of adoptively transferred adherent lymphokine-activated killer cells assessed by different cell labels.. Cancer Immunol Immunother.

[OCR_00736] Basse P., Hokland P., Hokland M. (1990). Comparison between 125IUdR and 51Cr as cell labels in investigations of tumor cell migration.. Int J Rad Appl Instrum B.

[OCR_00752] Bassil B., Dosoretz D. E., Prout G. R. (1985). Validation of the tumor, nodes and metastasis classification of renal cell carcinoma.. J Urol.

[OCR_00756] Bergers J. J., Den Otter W., Dullens H. F., De Groot J. W., Steerenberg P. A., Filius P. M., Crommelin D. J. (1994). Effect of immunomodulators on specific tumor immunity induced by liposome-encapsulated tumor-associated antigens.. Int J Cancer.

[OCR_00762] Brown N. J., Reed M. W. (1997). Leucocyte interactions with the mouse cremaster muscle microcirculation in vivo in response to tumour-conditioned medium.. Br J Cancer.

[OCR_00767] Ettinghausen S. E., Lipford E. H., Mulé J. J., Rosenberg S. A. (1985). Recombinant interleukin 2 stimulates in vivo proliferation of adoptively transferred lymphokine-activated killer (LAK) cells.. J Immunol.

[OCR_00772] Ettinghausen S. E., Rosenberg S. A. (1986). Immunotherapy of murine sarcomas using lymphokine activated killer cells: optimization of the schedule and route of administration of recombinant interleukin-2.. Cancer Res.

[OCR_00776] Fukumura D., Salehi H. A., Witwer B., Tuma R. F., Melder R. J., Jain R. K. (1995). Tumor necrosis factor alpha-induced leukocyte adhesion in normal and tumor vessels: effect of tumor type, transplantation site, and host strain.. Cancer Res.

[OCR_00788] Hayat K., Rodgers S., Bruce L., Rees R. C., Chapman K., Reeder S., Dorreen M. S., Sheridan E., Sreenivasan T., Hancock B. W. (1991). Malignant melanoma and renal cell carcinoma: immunological and haematological effects of recombinant human interleukin-2.. Eur J Cancer.

[OCR_00794] Jain R. K. (1988). Determinants of tumor blood flow: a review.. Cancer Res.

[OCR_00795] Lafreniere R., Rosenberg S. A. (1985). Successful immunotherapy of murine experimental hepatic metastases with lymphokine-activated killer cells and recombinant interleukin 2.. Cancer Res.

[OCR_00800] Ley K., Bullard D. C., Arbonés M. L., Bosse R., Vestweber D., Tedder T. F., Beaudet A. L. (1995). Sequential contribution of L- and P-selectin to leukocyte rolling in vivo.. J Exp Med.

[OCR_00810] Melder R. J., Salehi H. A., Jain R. K. (1995). Interaction of activated natural killer cells with normal and tumor vessels in cranial windows in mice.. Microvasc Res.

[OCR_00805] Melder R. J., Walker E. R., Herberman R. B., Whiteside T. L. (1990). Surface characteristics, morphology, and ultrastructure of human adherent lymphokine-activated killer cells.. J Leukoc Biol.

[OCR_00815] Murphy G. P., Hrushesky W. J. (1973). A murine renal cell carcinoma.. J Natl Cancer Inst.

[OCR_00819] Palmer P. A., Vinke J., Evers P., Pourreau C., Oskam R., Roest G., Vlems F., Becker L., Loriaux E., Franks C. R. (1992). Continuous infusion of recombinant interleukin-2 with or without autologous lymphokine activated killer cells for the treatment of advanced renal cell carcinoma.. Eur J Cancer.

[OCR_00827] Reed M. W., Wieman T. J., Schuschke D. A., Tseng M. T., Miller F. N. (1989). A comparison of the effects of photodynamic therapy on normal and tumor blood vessels in the rat microcirculation.. Radiat Res.

[OCR_00832] Rosenberg S. A., Lotze M. T., Muul L. M., Chang A. E., Avis F. P., Leitman S., Linehan W. M., Robertson C. N., Lee R. E., Rubin J. T. (1987). A progress report on the treatment of 157 patients with advanced cancer using lymphokine-activated killer cells and interleukin-2 or high-dose interleukin-2 alone.. N Engl J Med.

[OCR_00844] Rosenberg S. A., Packard B. S., Aebersold P. M., Solomon D., Topalian S. L., Toy S. T., Simon P., Lotze M. T., Yang J. C., Seipp C. A. (1988). Use of tumor-infiltrating lymphocytes and interleukin-2 in the immunotherapy of patients with metastatic melanoma. A preliminary report.. N Engl J Med.

[OCR_00849] Sasaki A., Jain R. K., Maghazachi A. A., Goldfarb R. H., Herberman R. B. (1989). Low deformability of lymphokine-activated killer cells as a possible determinant of in vivo distribution.. Cancer Res.

[OCR_00854] Sasaki A., Melder R. J., Whiteside T. L., Herberman R. B., Jain R. K. (1991). Preferential localization of human adherent lymphokine-activated killer cells in tumor microcirculation.. J Natl Cancer Inst.

[OCR_00859] Schwarz R. E., Vujanovic N. L., Hiserodt J. C. (1989). Enhanced antimetastatic activity of lymphokine-activated killer cells purified and expanded by their adherence to plastic.. Cancer Res.

[OCR_00864] Wiltrout R. H., Gorelik E., Brunda M. J., Holden H. T., Herberman R. B. (1983). Assessment of in vivo natural antitumor resistance and lymphocyte. Migration in mice: comparison of 125I-iododeoxyuridine with 111indium-oxine and 51chromium as cell labels.. Cancer Immunol Immunother.

[OCR_00872] Wu N. Z., Klitzman B., Dodge R., Dewhirst M. W. (1992). Diminished leukocyte-endothelium interaction in tumor microvessels.. Cancer Res.

